# Two Novel *FBN2* Variants Causing Congenital Contractural Arachnodactyly

**DOI:** 10.1155/genr/8521542

**Published:** 2026-06-16

**Authors:** Juan Zhao, Xiaolan Zhong, Lijun Du, Shaoqing Jiang, Ping Fang, Kexin Ruan, Sujian He, Guanghong Li, Hui Li, Haiming Yuan

**Affiliations:** ^1^ Huadu District People’s Hospital of Guangzhou, Guangzhou, Guangdong, China; ^2^ The Third School of Clinical Medicine, Southern Medical University, Guangzhou, Guangdong, China, fimmu.com; ^3^ Department of Clinical Medicine, Gannan Medical University, Ganzhou, Jiangxi, China, gmu.cn; ^4^ Department of Medical Genetics, Dongguan Maternal and Child Health Hospital, Dongguan, Guangdong, China

**Keywords:** congenital contractural arachnodactyly, connective tissue, *FBN2*, genetic counseling, novel variants

## Abstract

Congenital contractural arachnodactyly (CCA) is a rare, autosomal dominant connective tissue disease characterized by arachnodactyly, camptodactyly, multiple joint contractures, tall and slender habitus, crumpled ears, and scoliosis. It shares overlapping features with Marfan syndrome (MFS). This condition is caused by pathogenic variants in the fibrillin 2 (*FBN2*) gene. Currently, approximately 200 variants in *FBN2* have been identified, with most of the variants located in the middle region of the gene (Exons 24–35). Here, we investigated the genetic etiology of CCA in two unrelated Chinese families. Whole‐exome sequencing (WES) identified a novel in‐frame deletion variant, NM_001999.4: c.4195_4209del, p.Trp1399_Gly1403del, in Exon 32 of *FBN2* that was detected in all 3 affected patients but absent in 6 unaffected family members. The other novel missense variant in Exon 27 of *FBN2* (c.3521G > A, p.Cys1174Tyr) was identified in an 8‐month‐old female patient who was diagnosed with CCA. This variant was verified to be inherited from her unaffected mother with low‐level mosaicism. Our study expands the mutation spectrum of *FBN2* and provides insights into the genotype–phenotype relationship in CCA as well as a foundation for its genetic diagnosis, counseling, and management.

## 1. Introduction

Congenital contractural arachnodactyly (CCA; MIM#121050), also named Beals syndrome, is an autosomal dominant connective tissue disease. The typical clinical manifestations include congenital arachnodactyly, large joint contractures, kyphoscoliosis and crumpled ears [1]. Other clinical phenotypes described a tall, slender stature, micrognathia, high‐arched palate, pectus carinatum, talipes equinovarus, and muscle hypoplasia [2–4]. CCA is caused by pathogenic variants in the fibrillin‐2 (*FBN2*) gene, sharing overlapping features with Marfan syndrome (MFS) linked to the fibrillin‐1 (*FBN1*) gene. Currently, approximately 1800 pathogenic variants in *FBN1* have been identified to be distributed throughout the gene ([5]; HGMD database). However, only 200 variants in *FBN2* observed in individuals with CCA cluster in a limited region of the gene, from Exons 24–35, which is homologous to the region of *FBN1* that harbors most pathogenic variants that lead to the neonatal MFS phenotype. Thus, this region is called the neonatal region [6–8]. Thus, it is necessary to collect more individuals with CCA to enrich the mutation spectrum of *FBN2.*

Here, we reported two unrelated Chinese families who displayed characteristics typical of CCA. Whole‐exome sequencing (WES) identified a novel in‐frame deletion variant, c.4195_4209del, p.Trp1399_Gly1403del, in Exon 32 of *FBN2* cosegregating in a four‐generation Chinese family with CCA. The other novel missense variant in Exon 27 of *FBN2* (c.3521G > A, p.Cys1174Tyr) was identified in an 8‐month‐old female patient with CCA, which was inherited from her unaffected mother with low‐level mosaicism. Our results expand the variant spectrum of *FBN2* and provide significant help for genetic diagnosis, counseling, and management for the affected families.

## 2. Materials and Methods

### 2.1. Ethical Compliance

This study was approved by the Ethics Committee of Huadu District People’s Hospital of Guangzhou (2021113). Written informed consent was obtained from the patient’s legal guardian for the publication of any potentially identifiable images or data included in this article.

### 2.2. WES and Sanger Sequencing

Genomic DNA was isolated via commercial nucleic acid extraction kits per the manufacturer’s protocol. WES was performed on an Illumina NovaSeq 6000 platform (Illumina, San Diego, CA, USA) to identify genetic variants in the patient cohort. Raw sequencing data were converted to FASTQ files with bcl2fastq2 (v2.20), aligned to the GRCh37/hg19 reference genome using BWA (v0.2.10) under default parameters, and variant calling was conducted via GATK (v3.7) HaplotypeCaller. Alignments were visualized in IGV. Common variants were filtered using the Genome Aggregation Database (gnomAD) and internal population frequency data. Candidate variants were validated by Sanger sequencing, with pathogenicity interpreted according to the ACMG/AMP guidelines [9].

## 3. Results

### 3.1. Case Presentation

#### 3.1.1. Family 1

**FIGURE 1:**
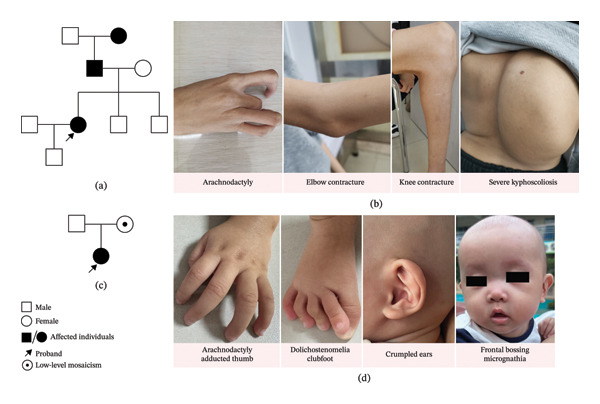
Pedigree of two families and clinical phenotypes of the probands. (a) Pedigree of Family 1 with cosegregation of the identified *FBN2* variant. (b) The proband showed arachnodactyly, camptodactyly, dolichostenomelia, contractures of the wrists, elbows and knees, and severe kyphoscoliosis. (c) Pedigree of Family 2. (d) The proband showed arachnodactyly, camptodactyly, adducted thumbs, dolichostenomelia, clubfeet, frontal bossing, crumpled ears, and micrognathia. Square: male; round: female; arrow: proband; filled: affected individuals; black dot: low‐level mosaicism.

The nonconsanguineous Chinese pedigree of CCA consists of 9 family members across four generations (Figure 1a). The proband was a 22‐year‐old female patient who presented with arachnodactyly, camptodactyly, dolichostenomelia, pectus deformity, and contractures of the wrists, elbows, and knees. Spinal evaluation revealed severe kyphoscoliosis in the thoracic and thoracolumbar regions (Figure 1b). She had a slender habitus but short stature (141 cm) secondary to severe kyphoscoliosis, with a body weight of 32.5 kg. Generalized muscle hypoplasia was noticed. The patient met the clinical diagnostic criteria for CCA according to the clinical scoring system for CCA [3] (Table 1). Her father and paternal grandmother displayed the same clinical features including arachnodactyly, camptodactyly, dolichostenomelia, multiple joint contractures, kyphoscoliosis, generalized muscle hypoplasia, and a short, slender habitus. No other pathological features, such as crumpled ears, cardiovascular, neurological, or ocular abnormalities, were observed in the proband or other affected family members. She had a healthy child. Intrafamilial phenotypic variability was modest.

**TABLE 1:** Clinical scores for our patients with CCA.

Clinical feature	Patient 1	Patient 2
Crumpled ears (3 points)	−	+
Arachnodactyly (3 points)	+	+
Camptodactyly (3 points)	+	+
Contractures of large joints (3 points)	+	+
Pectus deformity (2 points)	+	−
Dolichostenomelia (2 points)	+	+
Kyphoscoliosis (1 point)	+	−
Muscle hypoplasia (1 point)	+	+
Highly arched palate (1 point)	−	−
Micrognathia (1 point)	−	+
Scores	15	16

#### 3.1.2. Family 2

The female patient was the first‐born child of a Chinese nonconsanguineous healthy couple (Figure 1c). She was delivered vaginally spontaneously at 39 weeks of gestational age, with normal birth measurements: weight 3.2 kg, length 50 cm, and head circumference 34 cm. She was referred to the clinic at 40 days of age due to skeletal anomalies, presenting with arachnodactyly, camptodactyly, dolichostenomelia, adducted thumbs, and clubfeet. Obvious frontal bossing, crumpled ears, and micrognathia were observed (Figure 1d). She presented with impaired extension of the elbows and knees accompanied by large‐joint contractures. The patient was initially suspected of MFS. There was no family history of cardiovascular diseases or MFS in any family members. Subsequently, a detailed cardiovascular assessment (including aortic root dilatation, aortic regurgitation, mitral valve prolapse, and mitral regurgitation) and ocular examination for ectopia lentis and myopia showed unremarkable findings in both organ systems, which represent two cardinal features of MFS. Accordingly, the patient’s manifestations failed to meet the clinical diagnostic criteria for MFS based on the revised Ghent nosology, which requires the involvement of two cardinal features of MFS with minor involvement of a third organ system [10]. At 8 months of age, she had a slender stature, with a height of 70 cm and a weight of 6.3 kg (< −2 SD). She could not sit independently for a prolonged period, indicating muscle hypoplasia. No scoliosis or kyphoscoliosis was observed at this age. After a comprehensive assessment, the patient was clinically diagnosed with CCA in accordance with the clinical scoring system for CCA [3] (Table 1). She showed no aberrant cognitive development, and no signs of cardiovascular abnormalities or ocular complications were detected. Her joint contractures showed partial improvement following special care, including daily physical massage and passive joint extension.

### 3.2. Genetic Analysis

**FIGURE 2:**
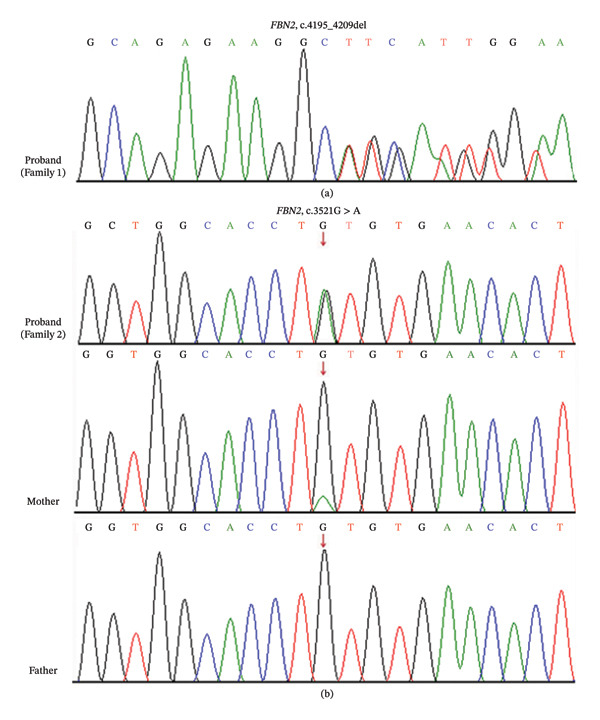
Variant identification by Sanger sequencing. An in‐frame deletion variant in *FBN2* (c.4195_4209del, p.Trp1399_Gly1403del) was detected in the proband (a). A missense *FBN2* variant (c.3521G > A, p.Cys1174Tyr; red arrow) was detected in the proband, was absent from the father, and was inherited from the unaffected mother with low‐level mosaicism (b).

WES identified a novel in‐frame deletion variant in *FBN2* (c.4195_4209del, p.Trp1399_Gly1403del) in the proband of Family 1, which was validated by Sanger sequencing, and the primer sequences are listed in Table 2 (Figure 2a). The variant was inherited from the proband’s father and paternal grandmother (both with matching phenotypes) and cosegregated with CCA across the four‐generation Chinese family, with no unaffected members carrying it. At 17 weeks’ gestation, prenatal Sanger sequencing confirmed that the fetus was a noncarrier, and the child showed no CCA clinical features at 3 years of age. Mapping to the 18th calcium‐binding epidermal growth factor‐like motif (cbEGF18) of *FBN2*, the variant was absent from the gnomAD and the 1000 Genomes Project. According to the ACMG/AMP guidelines, it was classified as clinically pathogenic, thereby underlying the clinical manifestations of affected individuals [9].

**TABLE 2:** Primers for Sanger sequencing validation of *FBN2* variants.

*FBN2* variants	Primer sequences
c.4195_4209del, p.Trp1399_Gly1403del	Forward 5′‐CCAAGTCCTCTTGGAGCCTA‐3′
	Reverse 5′‐AAACAGGTGCCAAGGCATAC‐3′

c.3521G > A, p.Cys1174Tyr	Forward 5′‐CCCTTGTGAGCCTGCTGATTTC‐3′
	Reverse 5′‐GATGGGAAGACGCTGGGCAAAG‐3′

A novel missense variant, c.3521G > A, p.Cys1174Tyr, in *FBN2* was detected in the female patient in Family 2. This variant was inherited from the unaffected mother with low‐level mosaicism (11%), which was confirmed by familial Sanger sequencing, and the primer sequences are listed in Table 2 (Figure 2b). This variant occurred in the highly conserved the 13th cbEGF motif (cbEGF13), and the p.Cys1174Tyr substitution was predicted to remove a conserved cysteine residue required for the correct folding of the domain. This variant was not present in either the gnomAD or the 1000 Genomes Project and was predicted to be damaging by multiple *in silico* tools (MutationTaster [Deleterious], SIFT [Deleterious]). Thus, the variant was assessed as clinically pathogenic according to the ACMG/AMP guidelines [9].

## 4. Discussion

The *FBN2* gene is located on 5q23.3, consists of 65 exons, and encodes *FBN2*, an elastin‐associated microfibrillar protein composed of 2912 amino acids. The protein contains an N‐terminal signal peptide, followed by 3 tandem N‐terminal epidermal‐growth‐factor (EGF)–like domains and 43 calcium‐binding EGF‐like (cbEGF) repeats interspersed with another EGF‐like domain, a glycine‐rich region, 7 TGFβ binding protein‐like (TB) domains, and two hybrid domains [11]. *FBN2* protein is an essential component of connective tissue microfibrils and participates in elastic fiber assembly. Microfibrils serve as a scaffold for elastin deposition and modification during elastic fiber formation in the extracellular matrix (ECM), which is very important in elastic, highly dynamic tissues. Microfibrils play a vital role in maintaining tissue integrity (such as elasticity and flexibility) and regulating the bioavailability of extracellular cytokines, thus mediating cell signaling, which is crucial for tissue homeostasis and remodeling [12–14]. Pathogenic variants in *FBN2* may affect signaling properties and the structural integrity of mature microfibrils, eventually disrupting the function of connective tissue and causing CCA, a very rare autosomal dominant connective tissue disorder [15, 16].

In this study, we investigated 2 pedigrees with 4 individuals diagnosed with CCA, based on their clinical and molecular profiles. In Family 1, a novel in‐frame deletion variant (c.4195_4209del, p.Trp1399_Gly1403del) in Exon 32 of *FBN2* was identified in all 3 CCA patients but was absent in 6 healthy members. Thus, this variant was obviously cosegregated in the four‐generation pedigree. The patients displayed arachnodactyly, camptodactyly, dolichostenomelia, joint contractures, kyphoscoliosis, and muscle hypoplasia, which met the clinical diagnostic criteria for CCA according to the established clinical scoring system [3]. Crumpled ears were the common feature of CCA but were not observed in the affected individuals in this family. It has been reported in the literature that crumpled ears become less pronounced over time [17, 18]. In contrast to the tall and slender habitus described in most CCA patients, the patients in this study displayed an obvious short and slender habitus. This difference may be attributed to progressive and severe kyphoscoliosis in our patients. Scoliosis, one of the most severe complications of CCA, occurs in approximately half of patients, tends to progress over time, and requires regular follow‐up and even surgical treatment [19]. Our patients received appropriate interventions but not surgical treatment, eventually resulting in severe kyphoscoliosis. Thus, they displayed a short and slender stature. Joint contractures are typical features of CCA and ameliorate in most cases, with potential benefits from physiotherapy [20]. However, our patients still presented with severe joint contractures, with unnoticeable intrafamilial variability. Thus, our patients carrying this variant appeared to display severe phenotypes of CCA. Certainly, additional cases with the same variant need to be collected to verify this conclusion.

In Family 2, a novel missense variant in Exon 27 of *FBN2* (c.3521G > A, p.Cys1174Tyr) was identified in an 8‐month‐old female patient, which was inherited from her unaffected mother with low‐level mosaicism. She displayed typical features of CCA, including crumpled ears, arachnodactyly, camptodactyly, large joint contractures, dolichostenomelia, muscle hypoplasia, and micrognathia. Early genetic counseling was provided for this family, and a molecular diagnosis was obtained for the female patient at 2 months of age. Since then, she was given daily physical therapy, and her joint contractures were obviously resolved. However, she could only sit independently for a short period, suggesting persistent muscle hypoplasia. Active rehabilitation, adequate nutrition, and further long‐term follow‐up are required for this patient.

**FIGURE 3:**
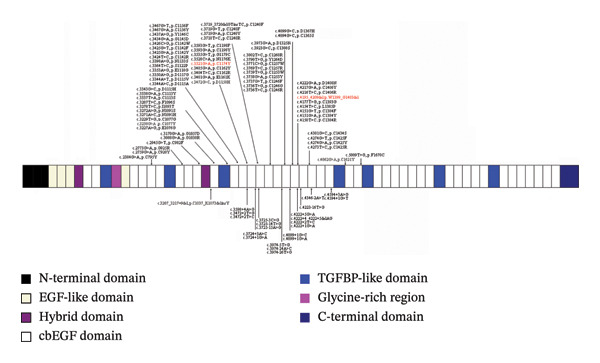
Schematic representation of the *FBN2* protein domains and positions of the identified variants. *FBN2* protein domains are shown as colored boxes relative to the size of the full‐length protein. Localization of variants identified is shown by dots. Black: variants identified in the literature. Red: novel variants identified in this study.

Subsequently, we systematically reviewed and analyzed variants in *FBN2*. Currently, more than 200 variants have been recorded in the literature, with the clinical significance of some variants remaining elusive [3, 21–23]. All variants in *FBN2* were manually assessed by our team according to the ACMG guidelines. A total of 93 pathogenic/likely pathogenic variants with sufficient evidence were identified, including the 2 novel variants revealed in our study. It was noticed that missense and splicing variants were the most common types, accounting for 75.3% (70/93) and 23.7% (22/93), respectively (Figure 3). The majority of the identified *FBN2* variant sites associated with CCA are located in the so‐called neonatal region (Exons 24–35), encoding a stretch of cbEGF‐like domains and playing a role in the stability and conformation of *FBN2*. A review of all published splicing variants revealed that these variants actually cause in‐frame deletions without altering the mRNA level of FBN2. It further supports the hypothesis that pathogenic variants in *FBN2* cause CCA through a dominant‐negative effect mechanism [3, 13, 15]. To date, no obvious mutation hotspots have been identified in *FBN2*. Certainly, it is also necessary to collect more cases with variants in *FBN2* to enrich the mutation spectrum of *FBN2.*

In conclusion, we identified two novel variants in *FBN2* in two unrelated Chinese families diagnosed with CCA. These variants expand the *FBN2* mutation spectrum and provide a sound basis for genetic diagnosis, counseling, and management of CCA.

**Nomenclature**
CCA: Congenital contractural arachnodactyly
FBN2: Fibrillin 2 gene
MFS: Marfan syndrome
WES: Whole‐exome sequencing
cbEGF: Calcium‐binding epidermal growth factor
EGF: Epidermal growth factor
SNVs: Single‐nucleotide variants
HGMD: Human Gene Mutation Database

## Author Contributions

Juan Zhao and Xiaolan Zhong drafted the first version of the manuscript. Haiming Yuan and Hui Li were responsible for the design of the project, data analysis, and revised the manuscript. Lijun Du and Shaoqing Jiang evaluated the clinical features of the patients in detail. Ping Fang and Kexin Ruan carried out the molecular studies.

Sujian He and Guanghong Li coordinated the clinical evaluation.

## Funding

This study was financed by Huadu District Basic and Applied Basic Research Joint Funding Project (District‐Hospital Collaboration), Guangzhou (Grant No. 23HDQYLH15).

## Disclosure

All the authors have read and approved the manuscript.

The funding body participated in the design, experimental operation, and result interpretation of the project.

## Ethics Statement

This study was approved by the Medical Ethics Committee of Huadu District People’s Hospital of Guangzhou. Written informed consent was obtained from the legal guardians for the publication of any potentially identifiable images or data included in this article.

## Consent

Consent to publish has been obtained from the guardians of all involved individuals.

## Conflicts of Interest

The authors declare no conflicts of interest.

## Data Availability

The data presented in the study are deposited in NODE (https://www.biosino.org/node) with the accession number OEP00006578 or accessible through the following URL: https://www.biosino.org/node/project/detail/OEP00006578.
